# TXNIP/TRX/NF-κB and MAPK/NF-κB pathways involved in the cardiotoxicity induced by Venenum Bufonis in rats

**DOI:** 10.1038/srep22759

**Published:** 2016-03-10

**Authors:** Qi-rui Bi, Jin-jun Hou, Peng Qi, Chun-hua Ma, Rui-hong Feng, Bing-peng Yan, Jian-wei Wang, Xiao-jian Shi, Yuan-yuan Zheng, Wan-ying Wu, De-an Guo

**Affiliations:** 1 Shanghai Research Center for Modernization of Traditional Chinese Medicine, National Engineering Laboratory for TCM Standardization Technology, Shanghai Institute of Materia Medica, Chinese Academy of Sciences, Haike Road 501, Shanghai 201203, China; 2 College of Traditional Chinese Medicine, China Pharmaceutical University, Nanjing 210009, China

## Abstract

Venenum Bufonis (VB) is a widely used traditional medicine with serious cardiotoxic effects. The inflammatory response has been studied to clarify the mechanism of the cardiotoxicity induced by VB for the first time. In the present study, Sprague Dawley (SD) rats, were administered VB (100, 200, and 400 mg/kg) intragastrically, experienced disturbed ECGs (lowered heart rate and elevated ST-segment), increased levels of serum indicators (creatine kinase (CK), creatine kinase isoenzyme-MB (CK-MB), alanine aminotransferase (ALT), aspartate aminotransferase (AST)) and serum interleukin (IL-6, IL-1β, TNF-α) at 2 h, 4 h, 6 h, 8 h, 24 h, and 48 h, which reflected that an inflammatory response, together with cardiotoxicity, were involved in VB-treated rats. In addition, the elevated serum level of MDA and the down-regulated SOD, CAT, GSH, and GPx levels indicated the appearance of oxidative stress in the VB-treated group. Furthermore, based on the enhanced expression levels of TXNIP, p-NF-κBp65, p-IκBα, p-IKKα, p-IKKβ, p-ERK, p-JNK, and p-P38 and the obvious myocardial degeneration, it is proposed that VB-induced cardiotoxicity may promote an inflammatory response through the TXNIP/TRX/NF-κB and MAPK/NF-κB pathways. The observed inflammatory mechanism induced by VB may provide a theoretical reference for the toxic effects and clinical application of VB.

Venenum Bufonis (VB, Chinese name “Chan Su”), a well-known Traditional Chinese Medicine, is the dried white extract of the auricular and skin glands of *Bufo gargarizans* Cantor or *Bufo melanostictus* Schneider^1^. It has been used traditionally for the treatment of deep-rooted carbuncles and boils, throat swelling and pain, coma, abdominal pain, heart stroke, vomiting, and diarrhea. In addition, it also serves clinically as an anti-cancer, anti-radiation, cardiotonic, diuretic, and anodyne agent^2^,^3^,^4^,^5^. The toxicity effects caused by VB have attracted considerable attention, especially the cardiotoxicity, which is a similar manifestation to the effects of digitalis. It is the most severe side effect threatening patients, and may induce bradycardia, atrioventricular conduction blockage, irreversible cardiomyopathy, and even sudden death^6^,^7^,^8^,^9^.

As is well-known, VB contains a variety of chemical components, including cardiotonic steroids (bufo steroids), indoleamines, fatty acids, polysaccharides, peptides, amino acids, and sterols^10^,^11^,^12^. Unfortunately, the main therapeutic ingredients of VB (bufalin, cinobufotalin, resibufogenin, and cinobufagin) are also the origin of its toxicity^13^, and are specific and potent inhibitors of Na^+^/K^+^ ATPase. To address the cardiotoxicity caused by VB, an increasing number of studies have been reported. *In vitro* studies indicated that the cardio-electrophysiological effects of VB could be due to inhibition of Na^+^/K^+^-ATPase^14^, alteration of calcium transients^15^, calcium overload^16^ and the redox modification of ryanodine receptors^17^. An *in vivo* metabolomics study demonstrated that the cardiac damage resulting from VB was associated with oxidative stress, mitochondrial dysfunction, and energy metabolism^18^. Although intensive investigations on VB-induced cardiotoxicity have been sustained for decades^19^,^20^, the potential mechanisms of these effects have not been fully elucidated. Considering that Na^+^/K^+^ ATPase has the functions as a signal transducer or integrator to regulate ROS^21^, and the Src/MAPK pathway^22^, as well as intracellular calcium^23^, both the oxidative stress and the inflammatory effects involved in a possible pathology were taken into consideration.

Thioredoxin-interacting protein (TXNIP), the endogenous inhibitor of thioredoxin (TRX), has been linked to oxidative stress and inflammation in a number of diseases, including atherosclerosis^24^, myocardial ischemia^25^, cancer^26^, and the degeneration of the central auditory system^27^. The MAPKs signaling pathway may play an important role in the development of cardiotoxicity^28^. Both TXNIP and MAPK can motivate NF-κB and active downstream genes to further modulate the inflammatory responses which would regulate the pathological state. Thus, the current study focused on the TXNIP/TRX/NF-κB and MAPK/NF-κB pathways to illustrate the inflammatory mechanism of the cardiotoxicity induced by VB, which could be of significance in developing an understanding of the toxic mechanism associated with VB.

## Results

### Effects of VB on the electrocardiogram (ECG)

**Figure 1:**
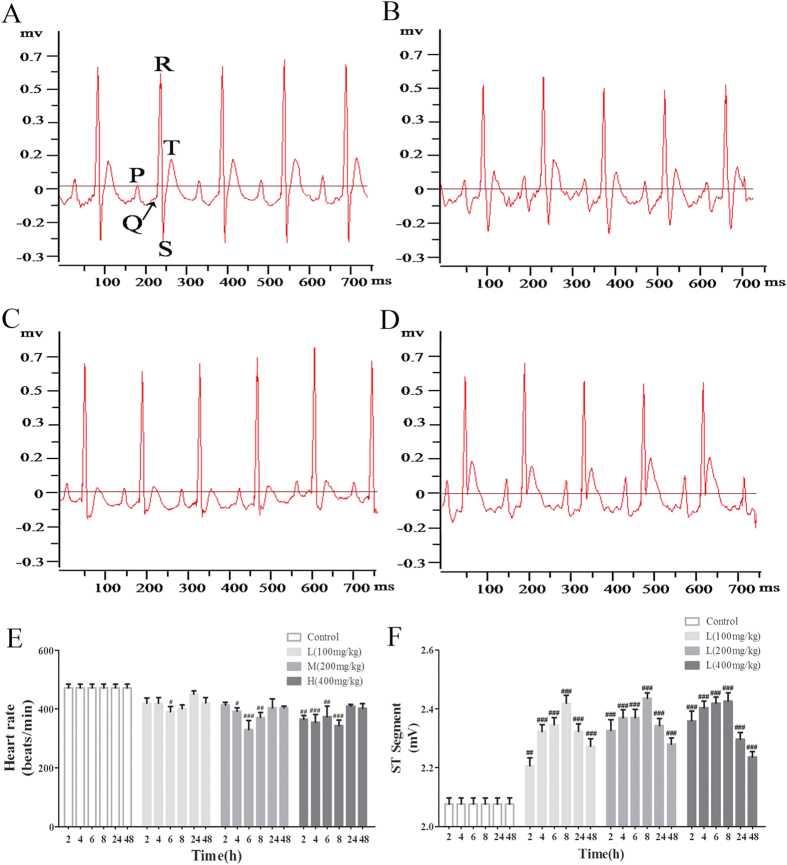
Effects of VB on electrocardiogram (ECG). The representative ECG tracing 4 h after administration of VB (**A–E**). (**A**) Control group; (**B**) 100 mg/kg group; (**C**) 200 mg/kg group; (**D**) 400 mg/kg group; The effects of VB on heart rate (**E**) and the ST-segment derivation (**F**) were statistically analyzed. All values given are the mean ± SD of eight rats for each group. ^#^p < 0.05, ^##^p < 0.01, and ^###^p < 0.001 vs. control group.

The ECGs of rats in the different group were recorded at 2 h, 4 h, 6 h, 8 h, 24 h, and 48 h. Figure 1A–D are representative figures at 4 h of the different groups. Rats in control group did not exhibit any changes in their ECG pattern (Fig. 1A), heart rate (Fig. 1E), and ST-segment derivation (Fig. 1F). Marked disturbances in ECG patterns (Fig. 1B–F) (e.g. elevation of ST-segment and decreased heart rate) were observed in the VB-treated groups.

### Effects of VB on body temperature

**Figure 2:**
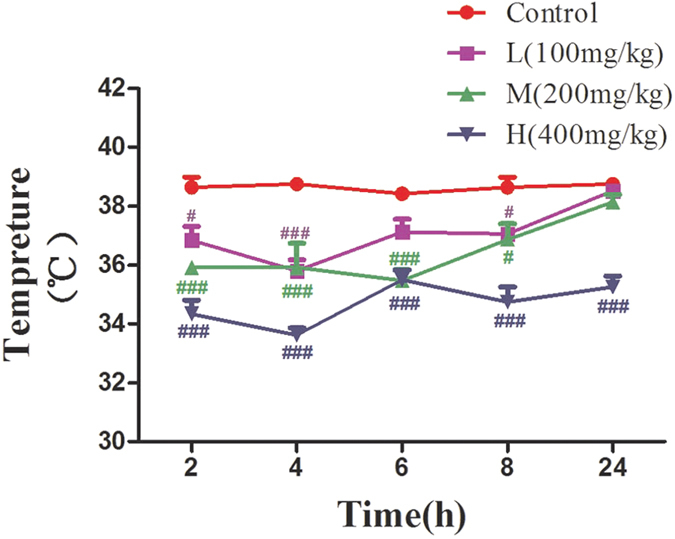
Effects of VB on body temperature. Body temperature was measured after the administration of VB (100 mg/kg, 200 mg/kg, and 400 mg/kg) at 2 h, 4 h, 6 h, 8 h, and 24 h. All values given are the mean ± SD of eight rats for each group. ^#^p < 0.05, ^##^p < 0.01, and ^###^p < 0.001 vs. control group.

Figure 2 shows the anal temperatures recorded during the experiment protocol. As can be seen, the significant reduction of temperature in the VB-treated groups (100 mg/kg, 200 mg/kg, and 400 mg/kg) displayed some degree of dose-dependence compared with the control group examined at 2 h, 4 h, 6 h, 8 h, and 24 h.

### Effects of VB on serum CK-MB, CK, AST, and ALT

**Figure 3:**
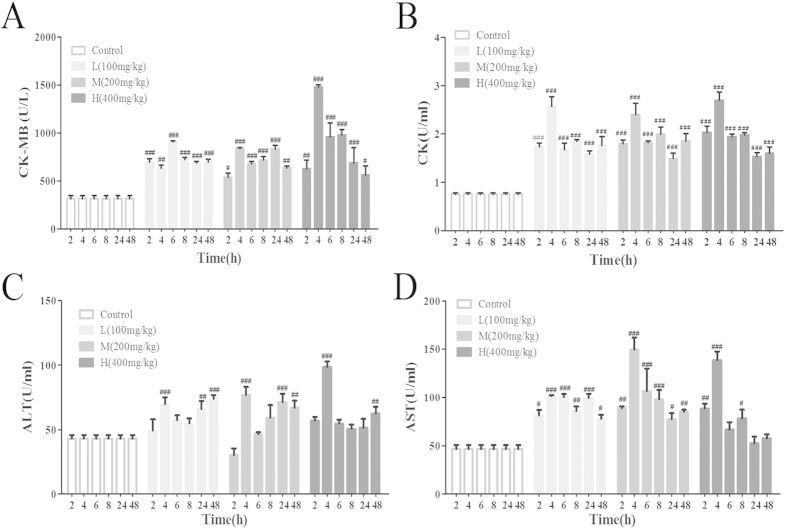
Effects of VB on serum CK-MB (**A**), CK (**B**), ALT (**C**), and AST (**D**). The level of serum CK-MB, CK, AST, and ALT in rats treated with VB (100 mg/kg, 200 mg/kg, and 400 mg/kg) at 2 h, 4 h, 6 h, 8 h, 24 h, and 48 h were test according to the manufacturer’s instructions. All values given are the mean ± SD of eight rats for each group. ^#^p < 0.05, ^##^p < 0.01, and ^###^p < 0.001 vs. control group.

The effects of VB on serum CK-MB, CK, AST, and ALT level are summarized in Fig. 3. The levels of CK-MB, CK, AST, and ALT in the VB-treated groups (100 mg/kg, 200 mg/kg, and 400 mg/kg) increased to the highest level at 4 h, indicating that the myocardium was damaged quickly after the administration of VB.

### Effects of VB on serum SOD, MDA, CAT, GSH, and GPx

**Figure 4:**
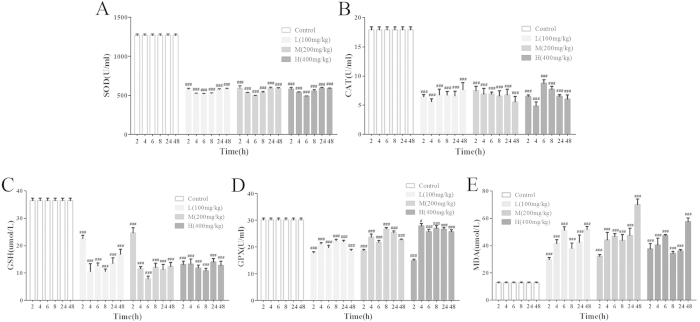
Effects of VB on serum SOD (**A**), CAT (**B**), GSH (**C**), GPx (**D**), and MDA (**E**). Rats were administered with different dosages (100 mg/kg, 200 mg/kg, and 400 mg/kg) of VB. SOD, CAT, GSH, GPx, and MDA were measured at 2 h, 4 h, 6 h, 8 h, 24 h, and 48 h. All values given are the mean ± SD. ^#^p < 0.05, ^##^p < 0.01, and ^###^p < 0.001 vs. control group (n = 8).

As shown in Fig. 4, rats treated with VB demonstrated an obvious inhibition of SOD, CAT, GSH, and GPx activity, which indicated the disturbed balance of oxidative stress. In addition, the continuing elevation of MDA, an end product of lipid peroxidation, indicated the involvement of free-radical-induced oxidative injury caused by VB administration.

### Effects of VB on cytokine levels

**Figure 5:**
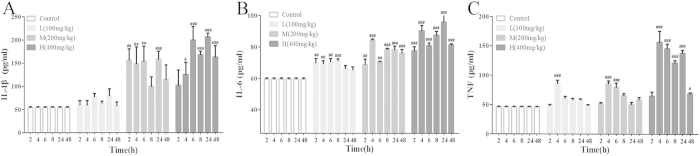
Effects of VB on IL-1β (**A**), IL-6 (**B**), and TNF-α (**C**). The serum pro-inflammatory cytokines IL-1β, IL-6, and TNF-α from different groups at 2 h, 4 h, 6 h, 8 h, 24 h, and 48 h were analyzed by ELISA kits. All values given are the mean ± SD. ^#^p < 0.05, ^##^p < 0.01, and ^###^p < 0.001 vs. control group (n = 8).

As shown in Fig. 5, pro-inflammatory cytokines IL-6, IL-1β, and TNF-α in serum showed different degrees of elevation after the various dosages, especially for the middle dosage and the high dosage group at 4 h, 6 h, 8 h, and 24 h in comparison with the control group according to Fig. 5. The observed up-regulation of IL-6, IL-1β, and TNF-α suggested that inflammatory responses had been induced in the VB-treated rats.

### Inflammatory mechanism study of the VB-treated rats

**Figure 6:**
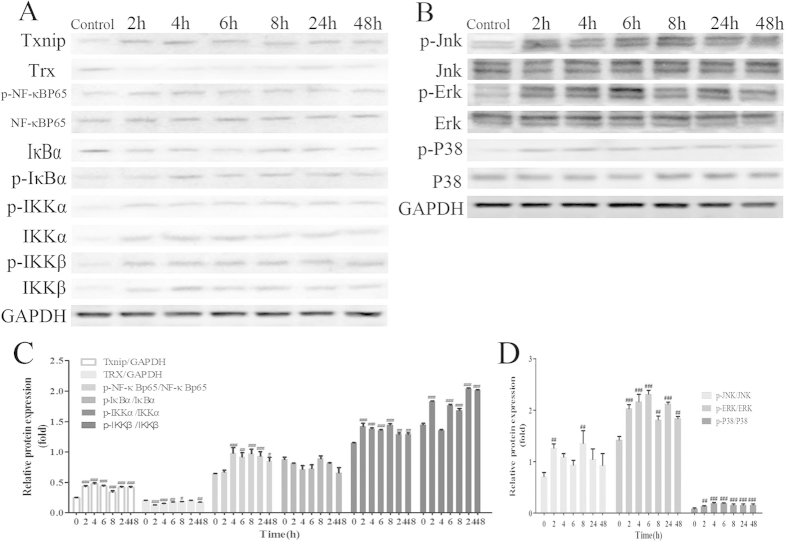
The effect of VB on the TXNIP/NF-кB pathway (**A**) and MAPK/NF-кB pathway (**B**). The expressions of related proteins from the TXNIP/NF-кB pathway and MAPK/NF-кB pathway were studied by Western blot analysis in VB-treated (400 mg/kg) rats. The quantification was performed based on the data of three separated experiments. All values given are the mean ± SD. ^#^p < 0.05, ^##^p < 0.01, and ^###^p < 0.001 vs. control group (n = 3).

**Figure 7:**
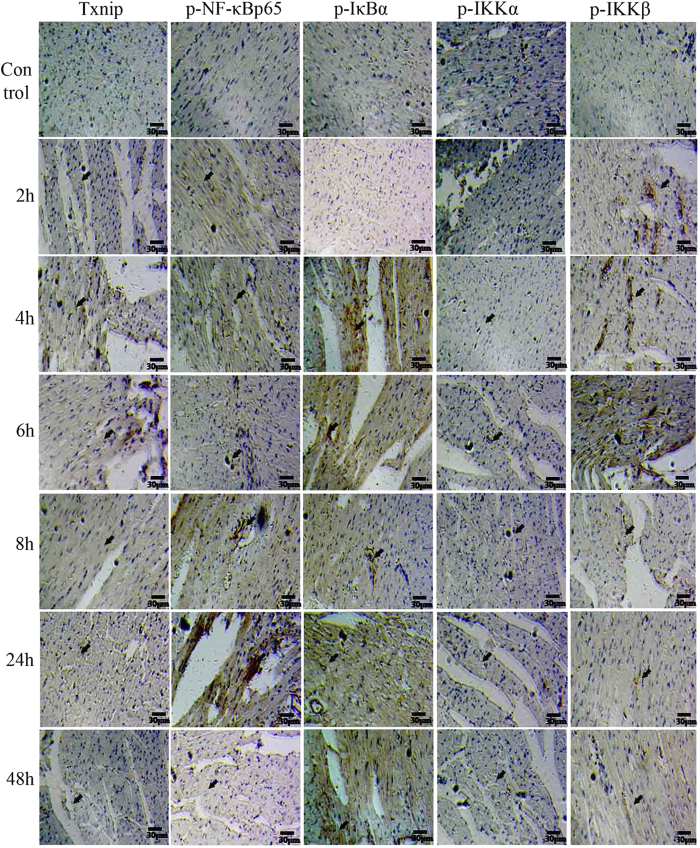
The effect of VB on the TXNIP/NF-кB pathway studied by immunohistochemistry analysis. The expression of TXNIP, p-NF-κBp65, p-IκBα, p-IKKα, and p-IKKβ of VB (400 mg/kg)-treated rats was investigated by immunohistochemical analysis.

The expressions of TXNIP, TRX, p-NF-κBp65, NF-κBp65, p-IκBα, IκBα, p-IKKα, IKKα, p-IKKβ, and IKKβ, p-ERK, ERK, p-JNK, JNK, p-P38, and P38 in the VB-treated rats (400 mg/kg) were assessed by Western blot analysis. For the TXNIP/TRX/NF-κB pathway, the increase of TXNIP, p-NF-κBp65, p-IKKα, p-IKKβ, and the decrease of TRX in heart tissue, were presented quite clearly. For the MAPK/NF-κB pathways, p-ERK, p-JNK, and p-P38 showed an apparent increase in levels (Fig. 6). Additionally, the enhanced expression of TXNIP, p-NF-κBp65, p-IκBα, p-IKKα, and p-IKKβ in VB(400 mg/kg)-treated rats were clearly observed at 4–48 h by immunohistochemistry (Fig. 7).

### Histopathological findings

**Figure 8:**
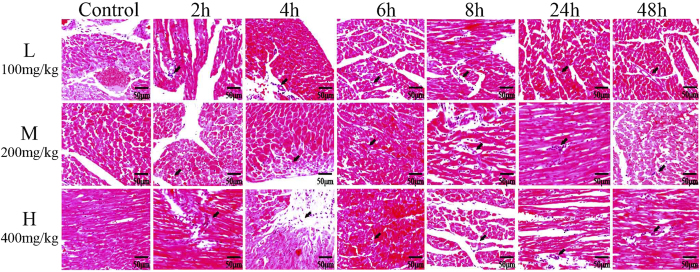
Effects of different VB treatment on myocardium histology. Representative histological changes in heart obtained from rats of different dosage groups (100 mg/kg, 200 mg/kg, and 400 mg/kg) under different times (2 h, 4 h, 6 h, 8 h, 24 h, and 48 h).

To further characterize the cardiotoxicity induced by VB, histopathological examination of heart tissues was conducted. Hearts from the control group animals exhibited regular cell distribution and normal myocardium architecture, while those harvested from the VB-treated groups displayed marked myocardial degeneration, including myofibrillar loss, cytoplasmic vacuolization, inflammatory cell infiltration, congestion, and edema (Fig. 8), which indicated the obvious heart damage.

## Discussion

Due to its various pronounced pharmacological effects, VB is widely used in several Asian countries (e.g. Laos, China, Japan, and India)^29^, and has been used clinically for the therapy of a wide range of diseases. However, the toxicity caused by VB limits its clinical application. Although there are an increasing number of reports drawing attention to the adverse cardiovascular effects associated with VB administraion, there is still little known about the mechanism of cardiotoxicity. Here, the TXNIP/TRX/NF-κB and MAPK/NF-κB pathways were intensively studied to investigate the mechanism of cardiotoxicity induced by VB in rats.

The cardiotoxicity effects in VB-treated rats can be observed directly through the readily-observed disturbances in the ECG. A certain degree of myocardial ischemia and arrhythmia can be deduced from the elevated ST-segment and decreased heart rate.

It was reported that body temperature is implicated in the isoprenaline-induced cardiotoxicity in rats^30^. In addition, hyperthermia was shown to be a feature of water-restraint stress-enhanced methamphetamine-induced cardiotoxicity^31^. Myocardial lesions are accompanied by changes in the thermoregulatory capacity of an animal, which can be monitored through alterations in body temperature directly. The data obtained in the present study revealed that different concentrations of VB effectively down-regulated the body temperature of the treated rats, indicating the possible cardiotoxic action of the drug.

Meanwhile, it was confirmed that AST, ALT, CK, and CK-MB, especially for CK-MB, which was released from the damaged myocytes, are the best markers for induced cardiotoxicity because of their tissue specificity^32^,^33^,^34^. The amount of these cellular enzymes present in the blood reflects the changes of serum membrane integrity and/or permeability^35^, which indicates certain types of heart damage, such as myocardial infarction, myocarditis, and heart failure. VB administration could lead to damage of the myocardial cell membrane or enhance its permeability, thereby exhibiting the significant elevation in the activities of serum CK-MB, CK, ALT, and AST in the current study. The increases in levels of these enzymes were in agreement with previous investigations^35^,^36^.

The pathogenesis of VB-induced cardiotoxicity is not entirely clear. Accumulating evidence indicates that oxidative stress plays a critical role in the pathogenesis of cardiotoxicity^37^,^38^. The heart tissue consists of postmitotic cells which intake fatty acids as the preferred substrate for energy supply, making it more susceptible to oxidative stress than other tissues^39^. The myocardium also possesses a set of antioxidant defense systems to prevent free radical invasion and to weaken their damaging effects. Antioxidant enzymes are recognized as the first guard of cell defense that protects the cellular ingredients against damage from oxidative stress. One of the main manifestations of cellular oxidative damage is lipid peroxidation of the myocardial membrane. In addition, as the most abundant non-protein thiol in the cell, GSH is considered to be the major cellular redox buffer. It is implicated in the scavenging of reactive oxygen species and the maintenance of membrane protein thiols. Furthermore, it serves as a substrate for GPx^40^. Briefly, SOD converts superoxide radicals into hydrogen peroxide, which is then subsequently catalyzed to water by CAT and GPx. In the present study, oxidative stress was observed in the VB-treated rats, as shown by the decreased antioxidant enzyme CAT and SOD activities, the depleted GSH and GPx levels, and the markedly elevated MDA. Therefore, it is speculated that VB increased the susceptibility of cardiomyocytes to reactive oxygen species, which might be attributed to the reduced activity of SOD and CAT. The level of lipid peroxidation end product, MDA, was markedly increased in the serum of VB-treated rats, which suggested the involvement of free-radical-induced oxidative cell membrane injury^41^.

Moreover, oxidative stress causes direct deleterious effects, and induces inflammatory responses through activation of the redox sensitive transcription factor NF-κB^42^ and the over-production of the pro-inflammatory cytokines IL-6, IL-1β, and TNF-α. As a critical mediator of inflammatory response, NF-κB could also regulate the expression of pro-inflammatory mediators that finally results in fundamental pathological changes in the form of cardiomyopathy, biventricular fibrosis, and transmural myocarditis^43^. In the current study, the serum pro-inflammatory cytokines IL-6, IL-1β, and TNF-α were remarkably up-regulated in the VB-treated group, indicating the potential toxicity of VB. It was assumed that VB might be a potential activator of NF-κB and could enhance the production of downstream inflammatory cytokines.

TXNIP, one of the reactive oxygen species-regulating factors, is related to the maintenance of TRX-mediated redox balance. As a unique protein that reduces thiols, TRX plays a vital role in the regulation of redox balance, cell growth^44^, and NF-κB activity. TRX functions as a promoter of NF-κB-dependent transcription by reducing the reactive thiol in the Rel DNA binding domain of the p50/p65 heterodimer^45^,^46^. NF-κB exists in the cytoplasm in an inactive form through combination with IκB inhibitory proteins. On stimulation, IκB is ubiquitinated and degraded by proteasome revealing the nuclear localization signaling of NF-κB, which promotes the transport of NF-κB from the cytoplasm to the nucleus and accelerates phosphorylation^38^. Interestingly, Hirota *et al.* suggested that over-expression of TRX in the nucleus enhances NF-κB activity, whereas the over-expression in the cytoplasm had the opposite effect, indicating that the upstream proteins of the NF-κB pathway might be suppressed by thiol reduction^47^. It was documented that TXNIP resultant TRX facilitates NF-κB activation and activates the inflammatory response in a model of acute lung injury^48^,^49^. Furthermore, an implication for the involvement of TXNIP in drug-induced oxidative injury was also reported^50^, which might be attributed to its effect on thioredoxin, one of the predominant cellular defense mechanisms against oxidative stress^51^, this requires further research. In this study, phosphorylation activated NF-κB p65 was found to be highly expressed in the VB-stimulated rats compared with the control group. Thus, it was proposed that the cardiotoxicity observed in the VB-treated group might be induced by inflammatory effects associated with the TXNIP/TRX/NF-κB pathway activated by oxidative stress.

Moreover, MAPKs, including ERK, JNK, and the p38 signaling pathway, are the key mediators to transform extracellular signals into cellular responses^52^. Diverse studies have shown that reactive oxygen species can also induce or mediate the activation of the MAPK pathways^53^. Activated p38-MAPKs are responsive to stress stimuli and are associated with cell apoptosis, differentiation and autophagy, which plays dual roles in myocardial ischemia-reperfusion injury^54^. Prior work had confirmed that the phosphorylation of p38 MAPK plays a vital role in doxorubicin-induced cardiotoxicity *in vitro*^55^. Specifically, p38 MAPK was demonstrated to be involved in the onset of cardiomyocytes apoptosis in ischemia-reperfusion-injured hearts^56^. Therefore, MAPKs in VB-treated rats were also studied to further explore the mechanism of cardiotoxicity. MAPK activation might act on upstream of the NF-κB pathway, since the inhibitors of MAPK activation have a negative effect on NF-κB activation^57^. The data obtained from the current study showed the increased expression of p-ERK, p-JNK, and p-P38, which was in agreement with previous investigations.

Collectively, the principal finding of this study demonstrated for the first time that the cardiotoxicity induced by VB is involved in inflammation, which might be correlated with TXNIP/TRX/NF-κB and MAPK/NF-κB pathways. Further investigation of VB-induced potential cardiotoxicity might improve the rational use of the drug and promote the development of novel approaches to address the clinical issues during therapy.

## Materials and Methods

### Main reagents and kits

Venenum Bufonis was purchased from Anhui Province and authenticated by Professor De-an Guo (Shanghai Institute of Materia Medica, Chinese Academy of Sciences). The voucher specimens were deposited at the Shanghai Research Center for Modernization of Traditional Chinese Medicine, Shanghai Institute of Materia Medica. The major constituents of VB were qualitatively and quantitatively analyzed, as shown in the supplementary information (see supplementary Fig. S1 and supplementary Table S1 online). TNF-α, IL-6, and IL-1β enzyme-linked immunosorbent assay (ELISA) kits were supplied by Nanjing KeyGEN Biotech. Co., Ltd. (Nanjing, China). CK-MB, CK, AST, ALT, MDA, SOD, CAT, GSH, and GPx kits were purchased from Jiancheng Bioengineering Institute (Nanjing, China). Primary antibodies against TXNIP, TRX, p-NF-κBp65, NF-κBp65, p-IκBα, IκBα, p-IKKα, IKKα, p-IKKβ, and IKKβ, p-ERK, ERK, p-JNK, JNK, p-P38, and P38 antibodies were produced by Cell Signaling Technology (Danvers, Massachusetts, USA).

All other chemicals and reagents used in the studies were of analytical grade and were purchased from approved organizations.

### Animals

Sprague Dawley (SD) rats (84 male and 84 female), weighing 180–220 g, were acquired from the Comparative Medicine Centre of Yangzhou University. They were housed in an animal center under standard laboratory conditions with a 12 h light/12 h dark cycle environment at 22–24 °C, humidity of 40–70%, and had free access to standard water and food pellets. All experimental procedures were carried out in strict accordance with the NIH Guidelines for the Care and Use of Laboratory Animals, and all protocols were approved by the Institutional Animal Care and Use Committee of the Shanghai Institute of Materia Medica. All surgery was performed under sodium pentobarbital anesthesia and all efforts were spared to ensure minimal animal suffering.

### Experimental protocols

According to the previous studies, the dose of VB administered to rats (Wistar rats and Sprague-Dawley rats) is between 100–500 mg/kg^58^,^59^,^60^. The previous acute toxicity study indicated that the LD_50_ was 786–800 mg/kg based on an ICR mouse model, which is equivalent to 550–560 mg/kg for rats in our laboratory. Dong *et al.* used 10-week old rats (240–300 g) administered intragastrically with a high dose of VB (500 mg/kg) because of the close positive relationship between toxicity tolerance and weight^58^. In this study, considering the weight variation between male and female rats, the use of 6-week old rats (weighing 180–220 g) was adopted. To minimize the occurrence of morbidity, the 400 mg/kg high dose was administered intragastrically. VB was ground into a 60-mesh powder and suspended in 0.5% carboxymethyl cellulose sodium salt (CMC-Na) aqueous solution.

The rats were randomly divided into seven groups (n = 24), each comprised of half males and half females as follows: control group, 2 h group, 4 h group, 6 h group, 8 h group, 24 h group, and 48 h group. Apart from the control group (n = 8) which were administered with 0.5% CMC-Na aqueous solution, each group consisted of three subgroups (n = 8) at the different doses of VB (100 mg/kg), VB (200 mg/kg), and VB (400 mg/kg).

### Recording of the electrocardiogram (ECG)

Rats were anaesthetized with sodium pentobarbital by intraperitoneal injection at 2 h, 4 h, 6 h, 8 h, 24 h, and 48 h after drug administration, then the ECG were recorded using the BL-420 S Biologic Function Experiment system (Chengdu, China), and the heart rate and ST-segment derivation were statistically analyzed.

### Effects of VB on body temperature

Anal temperatures of each group were recorded by electronic thermometer T15SL (Guangzhou Genial Technology Co., Ltd., Guangzhou, China) prior to the anesthesitic procedure.

### Sample preparation

After the rats were sacrificed, blood samples were collected from the carotid artery and centrifuged at 5000 rpm for 15 min. The supernatant were collected and set aside at −80 °C for analysis of biochemical parameters. In addition, hearts were harvested and stored at −80 °C waiting for Western blot analysis, immunohistochemical analysis, and histological studies.

### Measurement of the CK-MB, CK, AST, and ALT contents in serum

CK-MB, CK, AST, and ALT are important serum markers to assess myocardial function. Their activities were detected using the applicable ELISA kits according to the manufacturer’s instructions.

### Measurement of the SOD, CAT, GSH, GPx, and MDA contents in serum

The activity of SOD, GSH, GPx, and CAT, as well as the content of MDA, were determined using the applicable ELISA kits on the basis of the manufacturer’s instructions.

### Measurement of the cytokine content in serum

The concentrations of IL-6, IL-1β, and TNF-α in serum were analyzed by the applicable ELISA kits according to the manufacturer’s instructions.

### Western blot analysis

Myocardial tissues were minced and homogenized in ice-cold RIPA lysis buffer. Dissolved proteins were harvested and centrifuged at 12000 rpm for 5 min at 4  °C to remove the debris. The concentration of total protein was detected with the bicinchoninic acid (BCA) protein assay reagent. Equal amounts of protein were mixed with five-times loading dye (Laemmli Sample Buffer) and 2-mercaptoethanol, followed by heating at 95 °C for 5 min, they were then loaded onto SDS-polyacrylamide gel electrophoresis and transferred onto the polyvinylidene difluoride membrane. A blocking solution (5% non-fat milk in PBS) was used to incubate the sheets overnight at 4 °C using specific antibodies against TXNIP, TRX, p-NF-κBp65, NF-κBp65, p-IκBα, IκBα, p-IKKα, IKKα, p-IKKβ, IKKβ, p-ERK, ERK, p-JNK, JNK, p-P38, P38, and GAPDH. After washing with TBST three times, the bands were incubated for 1 h at room temperature with the corresponding secondary antibodies. Enhanced chemiluminescence detection (ECL) reagents and a gel imaging system (Tanon Science & Technology Co., Ltd., Shanghai, China) were used to visualize the membranes. Densitometric scanning of band intensities were performed by Quantity one (Bio-Rad, California, USA).

### Immunohistochemical analysis

Myocardial tissues were fixed in 10% (V/V) neutral buffered formalin for 48 h, embedded in paraffin wax, and cut into 4 μm thick slices. The paraffin sections were baked for 1 h in the oven, deparaffinized in xylene, rehydrated with graded ethanol solutions, microwaved in sodium citrate buffer, cooled to room temperature naturally and incubated in 3% hydrogen peroxide. Each section was blocked with 3% BSA at room temperature. After removing the blocking solution, sections were incubated with primary antibody overnight at 4 °C, The membrane was washed three times with TBST containing Tween 20, and incubated with horseradish peroxidase-conjugated secondary anti-rabbit antibodies for 1.5 h. After that, the samples were stained with DAB and then re-stained with hematoxylin. After dehydration and drying, the sections were mounted with neutral gum.

### Histological studies

Heart tissue sections were deparaffinized in xylene, processed with a graded ethanol series, and then stained with hematoxylin-Eosin (HE) solution for examination of the histopathology.

### Statistical analysis

All data were expressed as means ± standard deviation (SD). One-way analysis of variance (ANOVA) with the Tukey multiple comparison test was performed to evaluate the differences between groups. Figures were considered as statistically significant when *p* values < 0.05. Calculations were made using GraphPad Prism 5.0 (Graphpad, San Diego, CA, USA).

## Additional Information

**How to cite this article**: Bi, Q.- *et al.* TXNIP/TRX/NF-κB and MAPK/NF-κB pathways involved in the cardiotoxicity induced by Venenum Bufonis in rats. *Sci. Rep.* **6**, 22759; doi: 10.1038/srep22759 (2016).

## Supplementary Material

Supplementary Information
