# Supplementary material for: TXNIP/TRX/NF-κB and MAPK/NF-κB pathways involved in the cardiotoxicity induced by Venenum Bufonis in rats
Source: Sci Rep. 2016 Mar 10;6:22759. doi: 10.1038/srep22759 (PMC4785358; doi:10.1038/srep22759)
Supplement: Supplementary Information [file srep22759-s1.pdf]

TXNIP/TRX/NF-κB and MAPK/NF-κB pathways involved in the cardiotoxicity induced by Venenum Bufonis in rats

Qi-rui Bi[1,2,#], Jin-jun Hou[1,#], Peng Qi[1,#], Chun-hua Ma[2], Rui-hong Feng[1], Bing-peng Yan[1,2], Jian-wei Wang[1,2], Xiao-jian Shi[1,2], Yuan-yuan Zheng[1], Wan-ying Wu[1]*, and De-an Guo[1]*

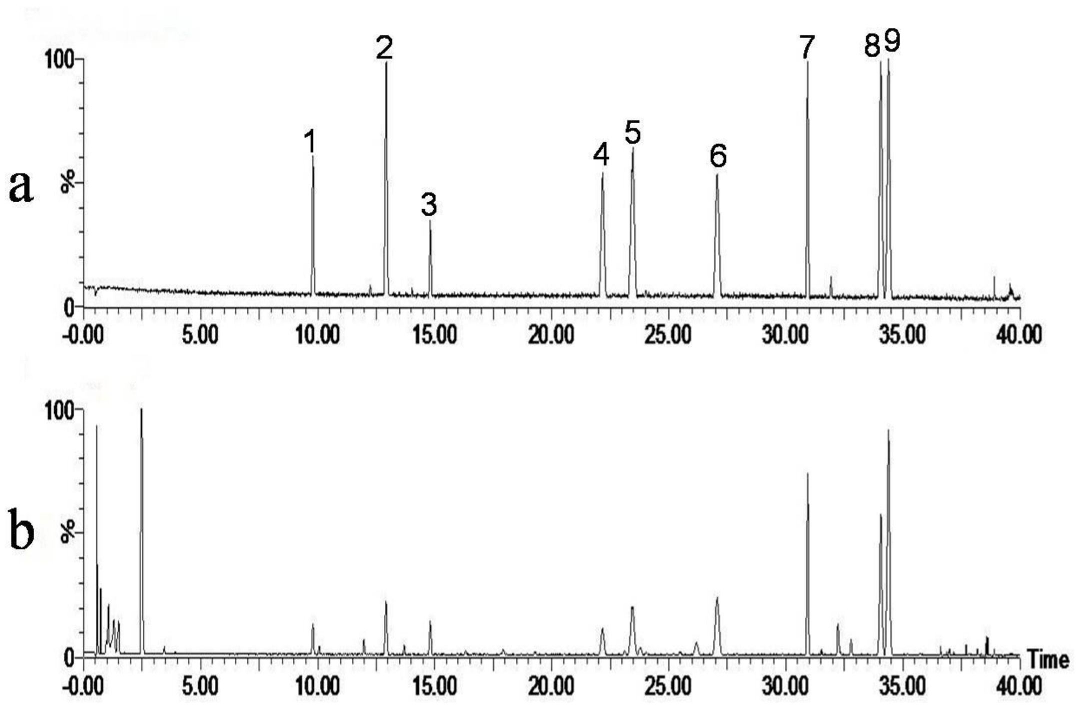

Supplyment figure S1. The Total ion chromatogram of mixed standard compounds and VB analyzed by UPLC-ESI-QTof. (a) Nine standard compounds: 1: desacetylbufotalin 2: arenobufagin 3: Hellebrigenin 4: telocinobufagin 5: bufotalin 6: cinobufotalin 7: bufalin 8: Resibufogenin 9: cinobufagin. (b) ESI-MS total ion current profile of VB methanol extract.

Supplement Table S1. The concentration of nine main compounds in VB methanol extract.

	RT	M+H	compounds	Content（%）
1	9.8	403	desacetylbufotalin	0.9
2	12.92	417	arenobufagin	0.97
3	14.81	417	Hellebrigenin	1.89
4	22.19	403	telocinobufagin	0.94
5	23.45	445	bufotalin	1.47
6	27.01	459	cinobufotalin	2.09
7	30.93	387	bufalin	3.13
8	34.06	385	Resibufogenin	2.23
9	34.39	443	cinobufagin	3.98
